# Orofacial Myofunctional Therapy: Investigating a Novel Therapeutic Approach for Pediatric Obstructive Sleep Apnea in Children with and Without Down Syndrome—A Study Protocol

**DOI:** 10.3390/children12060737

**Published:** 2025-06-06

**Authors:** Jolien Verbeke, Iris Meerschman, Karlien Dhondt, Els De Leenheer, Julie Willekens, Kristiane Van Lierde, Sofie Claeys

**Affiliations:** 1 Department of Rehabilitation Sciences, Centre for Speech and Language Sciences (CESLAS), Ghent University, Corneel Heymanslaan 10, 2P1, 9000 Ghent, Belgium; iris.meerschman@ugent.be (I.M.); kristiane.vanlierde@ugent.be (K.V.L.); 2 Department of Child & Adolescent Psychiatry, Pediatric Sleep Center, Ghent University Hospital, Corneel Heymanslaan 10, 9000 Ghent, Belgium; karlien.dhondt@ugent.be; 3 Department of Head and Skin, Ghent University Hospital, Corneel Heymanslaan 10, 9000 Ghent, Belgium; 4 Department of Otorhinolaryngology, Ghent University Hospital, Corneel Heymanslaan 10, 9000 Ghent, Belgium; els.deleenheer@ugent.be (E.D.L.); sem.claeys@ugent.be (S.C.); 5 Department of Pediatric Pulmonology, Ghent University Hospital, Corneel Heymanslaan 10, 9000 Ghent, Belgium; julie.willekens@uzgent.be; 6 Department of Speech-Language and Audiology, University of Pretoria, Pretoria 0002, South Africa

**Keywords:** obstructive sleep apnea, orofacial myofunctional therapy, oropharyngeal exercises, down syndrome, multidisciplinary research

## Abstract

Background/Objectives: Pediatric obstructive sleep apnea (OSA) is a prevalent medical condition, affecting 1–5% of non-syndromic children and 30–90% of children with Down syndrome. Given the severity of the condition and the associated health risks, early and effective treatment is crucial. However, current treatment modalities are often invasive or suffer from poor patient adherence. Additionally, adenotonsillectomy, the first-line treatment in pediatric OSA, seems not to be effective in every child, leaving children with residual OSA postoperatively. These challenges are particularly pronounced in high-risk populations, such as children with Down syndrome, highlighting the need for alternative therapeutic strategies. Therefore, a protocol is presented to evaluate the effectiveness of orofacial myofunctional therapy (OMT) as a treatment for OSA in two pediatric populations: (1) Non-syndromic children aged 4–18 years: 10 weeks of OMT. (2) Children with Down syndrome aged 4–18 years: 20 weeks of OMT. Effects of the OMT program will be evaluated on: sleep parameters (e.g., obstructive Apnea–Hyponea Index (oAHI), snoring frequency); orofacial functions (e.g., breathing pattern, tongue position at rest); quality of life outcomes. Methods: A pretest–posttest design will be used to evaluate the effectiveness of OMT in both children with and without Down syndrome and OSA. Both objective measures and patient-reported outcomes are being collected. Results: OMT is expected to improve orofacial functions, reduce OSA severity and symptoms, and enhance quality of life in both non-syndromic and syndromic children. Conclusions: This multidisciplinary research protocol, involving collaboration between ENT specialists and speech-language pathologists, aims to provide a comprehensive understanding of the potential benefits of OMT in treating OSA.

## 1. Introduction

### 1.1. Epidemiology and Therapeutic Challenges in Pediatric OSA

Obstructive Sleep Apnea (OSA) is a prevalent medical condition with a prevalence of 1% to 5% in otherwise healthy, non-syndromic children and 30% to 90% in children with Down syndrome [1,2]. OSA is classified under sleep breathing disorders and characterized by repeated episodes of upper airway (UA) obstruction due to UA collapse during sleep, with complete (apnea) or partial (hypopnea) interruption of airflow resulting in abnormal ventilation, abnormal breathing, and sleep disturbance [3]. The UA collapses are a result of increased UA collapsibility, anatomic narrowing of the UA, or both [1]. The underlying cause of pediatric OSA is considered multifactorial. Factors such as adenoid and tonsil hypertrophy, obesity, anatomical or neuromuscular deficits, and hypotonic neuromuscular disorders can be involved, leading to multilevel obstruction of the UA during sleep [4].

Among these factors, adenoid and tonsil hypertrophy is the most commonly reported cause of OSA in children, making adenotonsillectomy (AT) the standard therapeutic approach [4]. However, 20% of otherwise healthy, typically developing children suffer from residual OSA after AT [5]. The persistence of OSA post-AT can be attributed to the multifactorial etiology of OSA, suggesting that AT alone may not adequately address all underlying causes of airway obstruction. In children with Down syndrome, this challenge is even more pronounced. Despite undergoing AT, up to 55% of children with Down syndrome continue to experience residual OSA [2]. This high rate is due to the complex interplay of anatomical and functional predisposing factors for OSA in this population, including macroglossia, generalized hypotonia, mouth breathing, and narrow upper airways resulting from craniofacial abnormalities. These features contribute to increased UA collapsibility and multilevel obstructions, often limiting the effectiveness of single-treatment approaches such as AT [6,7,8].

Other treatment options for pediatric OSA include continuous positive airway pressure (CPAP) and rapid maxillary expansion (RME). These treatments also show several drawbacks in children, such as low adherence to CPAP and impaired facial growth due to wearing the mask and chin strap [4,9]. Furthermore, Guilleminault et al. [10] reported persistence or recurrence of OSA in nearly 65% of non-syndromic and non-overweight children several years after AT and RME. In children with Down syndrome, surgical procedures such as genioglossus advancement, lingual tonsillectomy, and tongue base volume reduction are described to reduce OSA [11]. However, many of these studies report a significant decrease in OSA post-surgery, but the Apnea–Hypopnea Index (AHI) still reveals severe residual OSA after intervention [12,13,14].

Untreated or inadequately treated OSA can lead to behavioral problems, school problems, hyperactivity, nocturnal enuresis, sleep terrors, insomnia, depression, and other psychiatric problems [3,4]. Compared to non-syndromic children, children with Down syndrome may be even more vulnerable to these effects of OSA [11]. The high prevalence of residual OSA post-AT and the negative impact of residual OSA on the overall health, highlight the importance of exploring new therapeutic modalities to treat OSA in children with and without Down syndrome.

### 1.2. Orofacial Myofunctional Disorders and Therapy in OSA

Recent research shows that orofacial myofunctional disorders (OMDs) such as mouth breathing, caudal tongue positioning, and genioglossus dysfunctions occur in patients with OSA [10,10,15]. These OMDs increase UA collapsibility and can therefore be associated with the occurrence of UA collapses and OSA [16]. One of the OMDs most frequently associated with OSA is mouth breathing [17]. Adenoid and tonsil hypertrophy, the main cause of pediatric OSA, narrows the nasopharynx and oropharynx [18]. If nasal resistance exceeds a certain level, a shift to mouth breathing occurs [19]. After removal of the obstructive tissue, more than half of the children maintain the habit of mouth breathing, which in turn is associated with the development of skeletal disorders, inflammation of pharyngeal tissues, and recurrence of OSA in the long term [10,10]. Lee, Guilleminault, Chiu, and Sullivan [10] found that non-syndromic children who were cured from OSA after AT but remained breathing through their mouth during the night showed a recurrence of OSA 12 months postoperatively. In addition, a significantly higher OSA severity post-AT (range 12–72 months) was found in children who did not undergo orofacial myofunctional therapy (OMT) post-AT compared to children who did undergo OMT post-AT to reduce mouth breathing [10,10].

Fitzpatrick, McLean, Urton, Tan, O’Donnell and Driver [16] explained the relationship between mouth breathing and OSA by stating that when the child opens the mouth during sleep, the tongue and the jaw make a posterior inferior movement. This movement results in a reduction in retroglossal and retropalatal diameter of the upper airway and prevents forceful contraction of the upper airway dilating muscles, which increases upper airway collapsibility and subsequently the frequency of OSA [20]. In addition, mouth breathing leads to a deactivation of nasal receptors, resulting in reduced maintenance of spontaneous ventilation and apneas [21]. Furthermore, the amount of nitric oxide (NO) released in the nose and paranasal sinuses, is decreased during mouth breathing. NO plays an important role in blood oxygenation and the maintenance of airway patency. Consequently, NO deficits induce upper airway collapsibility and OSA [22].

Other OMDs observed in patients with OSA are dysfunctions of intrinsic and extrinsic tongue muscles, such as the genioglossus muscle (GG) [10,15,23]. The GG is the main upper airway dilator muscle and is responsible for moving the tongue anteriorly, dilatating the oropharynx, and preventing upper airway obstruction [24,25]. A decreased ability of the GG to maintain airway patency during sleep causes repetitive upper airway closure [26]. Results of an electromyography study showed significantly greater GG activity during wakefulness and a greater decline of GG activity during sleep onset in non-syndromic children with OSA, indicating increased upper airway collapsibility [15]. Furthermore, one adult study found a significantly negative correlation between tongue protrusion strength and OSA severity (apnea-index). Kanezaki et al. [27] concluded that a higher tongue protrusion strength is associated with increased upper airway stabilization. No studies regarding tongue protrusion strength in children with OSA are available. However, one study examined upward tongue strength in 78 children with OSA, showing a significantly lower tongue strength in children with OSA compared to children without OSA [23].

Given the association between OMDs and OSA, OMT is emerging as a promising treatment for pediatric OSA in addition to or as a replacement for structural treatments such as AT [28]. OMT is a therapeutic method for neuromuscular rehabilitation programs consisting of a series of isotonic and isometric exercises, designed to enhance sensitivity, proprioception, mobility, coordination, and strength of the oropharyngeal structures involved in breathing, mastication, swallowing, and speech. Respiratory muscle training may be included as part of the therapy, aiming to strengthen pharyngeal, diaphragmatic, external intercostal, and accessory respiratory muscles. In patients with OSA, OMT aims to improve muscle resistance, balance the contraction of pharyngeal muscles, and correct abnormal functional and resting postures of the oropharyngeal structures in order to stabilize the upper airway during sleep [29,30].

Only a few small studies examined the effect of OMT as a secondary treatment for OSA in non-syndromic children after AT [10,10,31,32]. In these studies, OMT focused on the elimination of mouth breathing, the correction of tongue posture, and increasing tongue strength [31,32]. The results showed a significantly lower AHI, reflecting a decrease in OSA severity, in the groups that followed OMT [10,10,31,32]. However, the available literature about OMT as a secondary treatment for pediatric OSA is limited, and there is a lack of high-quality evidence. Studies show methodological limitations such as small sample sizes, non-standardized therapy methods, and limited outcome measurements [33]. Additionally, no studies examined the effect of OMT as primary treatment for pediatric OSA. When looking specifically at studies in children with Down syndrome, only one study investigated the effect of one week of OMT on OSA in this population [2]. Results showed a slight to negligible decrease in OSA, which is expected after only one week of therapy. No research is available regarding the effectiveness of long-term OMT on OSA in children with Down syndrome. Nevertheless, there is a major need for an alternative treatment to optimize health conditions and quality of life in this population [34].

## 2. Materials and Methods

### 2.1. Participants

#### 2.1.1. Non-Syndromic Children

Children with OSA will be recruited via the Pediatric Sleep Center, the Otorhinolaryngology and the Pneumology department of Ghent University Hospital. They will be selected to participate in the study based on the inclusion criteria: aged between 4 and 18 years, diagnosed with OSA confirmed by an oAHI > 1 on polysomnography. The lower age limit is chosen because a certain level of maturity is required before a child is capable to understand and perform OMT exercises [10]. Exclusion criteria are: history of OMT, orthodontic treatment in progress, other OSA treatments in progress, orofacial congenital deformities, nasal congestion, intellectual disability and obesity (>2 SD above P50). Both children with and without previous surgical removal of adenoids and/or tonsils will be included. Inclusion and exclusion criteria will be assessed through a questionnaire and an ENT examination.

#### 2.1.2. Children with Down Syndrome

Children with Down syndrome will be recruited via the Pediatric Sleep Center, the department of otorhinolaryngology, and the Down Clinic at Ghent University Hospital. They will be selected based on the inclusion criteria: aged between 4 and 18 years, diagnosis of Down syndrome (trisomy 21), diagnosis of residual OSA after adenoidectomy and/or tonsillectomy confirmed by polysomnography (oAHI >1). Exclusion criteria are: nasal congestion, inability to close the mouth and bring the tongue inside the dental arch, presence of other neuromuscular, craniofacial, and/or genetic disabilities, history of OMT, orthodontic treatment in progress, and other OSA treatments in progress. Inclusion and exclusion criteria will be assessed through a questionnaire and an ENT examination.

### 2.2. Sample Size

Based on a study of Villa, Evangelisti, Martella, Barreto and Del Pozzo [32], a sample size of *n* = 23 was calculated for the pretest–posttest study in non-syndromic children (G*Power, McNemar test, 2-sided). Calculation was based on the outcome ‘oral breathing’ with an α level of 0.005 (to account for multiple testing), a power of 0.80, and a decrease of 66.7% (12/18) in the proportion of children exhibiting oral breathing post-intervention. Taking into account a dropout rate of 15%, *n* = 27 will be the target in the study. For the pretest–posttest study in children with Down syndrome, sample size calculation is not yet possible given the innovative nature of this objective.

### 2.3. Design

Two separate pretest–posttest studies will be conducted to determine the effects of OMT in the management of pediatric OSA in non-syndromic children (pretest–posttest study 1) and children with Down syndrome (pretest–posttest study 2). Intervention will consist of a 10-week OMT in non-syndromic children and a 20-week OMT in children with Down syndrome [35]. Both studies include 2 measurement moments: (1) at the start of the study (baseline), and (2) after the therapy period. All measurement moments consist of an evaluation of orofacial myofunctional, sleep, and quality of life outcomes. To avoid observer bias, all assessments will be performed blindly, so assessors will be blinded to group allocation and study phase.

To reduce the number of therapy sessions and investigations in these children, no sham therapy will be used. Because of the complexity of OSA and the variability in patients within the objective, it is chosen not to compare the results against a control group but to look at performance at an individual level.

### 2.4. ENT Screening

At the start of the study, all children will undergo an Ear Nose Throat (ENT) examination to evaluate anatomy and functionality of the orofacial structures (Table 1). This initial assessment is crucial for understanding the baseline characteristics of the participants and determining any potential abnormalities that may impact the study outcomes. Table 1 provides an overview of the components of the ENT examination.

### 2.5. Demographic and Medical Questionnaire

At the start of the study, parents of children will be asked to fill out a questionnaire concerning medical (e.g., previous OSA treatments, timing of adenotonsillectomy) and demographic (e.g., sex, age, weight) information of their child. By recording these variables, the study aims to better understand variability in treatment outcomes and identify subgroups that may benefit most from the intervention.

### 2.6. Assessment Protocol

The effect of OMT in non-syndromic children (pretest–posttest study 1) and children with Down syndrome (pretest–posttest study 2) with OSA will be determined on (1) orofacial myofunctional, (2) sleep, and (3) Quality of Life (QoL) measures.

#### 2.6.1. Orofacial Myofunctional Assessment

Orofacial myofunctional outcomes will be evaluated during a perceptual and instrumental orofacial myofunctional assessment by 2 speech-language pathologists (SLPs) specialized in orofacial myofunctional disorders of Ghent University. These researchers will not be involved in the treatment and will be blinded for the study purposes.

#### 2.6.2. Oromyofunctional Postures, Mobility, and Functions

A perceptual orofacial myofunctional evaluation will be performed, using the Orofacial Myofunctional Evaluation with Scores (OMES) protocol, to evaluate posture (face, cheeks, tongue, lips, palate, maxilla/mandibula relation and mentalis muscle), mobility (lips, tongue, cheeks and jaw) and functions (breathing, deglutition and mastication) of the oropharyngeal structures [46]. Analysis of the OMES protocol is based on predefined ordinal rating scales and will be performed based on video recordings. Samples will be randomized and blindly evaluated by 2 specialized SLPs. The video recordings of the rater with the highest intra-rater reliability will be used for further analysis.

#### 2.6.3. Tongue and Lip Strength and Endurance

The IOWA Oral Performance Instrument (IOPI) (model 2.1; IOPI Medical LLC, Carnation, WA, USA) will be used to measure maximum lip and tongue strength and endurance following the instructions of Van Nuffelen et al. [47].

### 2.7. Sleep Assessment

#### 2.7.1. Screening for Pediatric Sleep Disorders

The Sleep Disturbance Scale for Children (SDSC) is a validated, parent-reported questionnaire to assess the presence of sleep disorders in children. Comprising 26 items on a 5-point Likert scale, the SDSC evaluates sleep disorders on six domains: disorders of initiating and maintaining sleep, sleep disordered breathing, disorders of arousal, sleep–wake transition disorders, disorders of excessive somnolence, and sleep hyperhidrosis [48,49]. Different types of sleep disorders can co-occur and symptoms can overlap among various pediatric sleep disorders. Therefore, it is important understand the child’s overall sleep health and identify potential co-occurring sleep disorders that may affect treatment outcomes.

#### 2.7.2. OSA Severity and Symptoms

All children will undergo a full-night laboratory polysomnography (PSG). The PSG will be performed and scored according to the standard criteria of the American Academy of Sleep Medicine by specialists from the Pediatric Sleep Center [46,47,50]. Electroencephalogram (EEG), chin electromyogram (EMG), and electrooculogram tracings will be used to determine sleep stage. In addition, PSG will provide data regarding respiratory events by measuring abdominal movements with strain gauges, oronasal airflow with an oronasal thermal sensor and nasal pressure transducer, and oxygen saturation with pulse oximetry. Snoring sounds will be detected by a snore microphone, and limb movements will be recorded using EMG [28,50]. PSG will objectively evaluate OSA severity (i.e., AHI, Arousal Index, Oxygen Desaturation Index, sleep efficiency) and OSA symptoms (i.e., snoring frequency). PSG results will be processed and interpreted by an expert at the Pediatric Sleep Center.

The Pediatric Sleep Questionnaire (PSQ) will be completed by the child’s parents to evaluate OSA symptoms. Twenty-two items within the following domains: snoring, observed apnea, daytime fatigue, and OSA-related behavioral disturbances will be scored on a dichotomous scale (present/absent). The variable obstructive Sleep-Related Breathing Disorder score (SRBD score) will be derived from the questionnaire by calculating the average of the non-missing items [51]. This questionnaire was proven to be reliable and valid with high sensitivity and specificity [52].

The Child Sleep Habits questionnaire (CSHQ) will be completed by the child’s parents to evaluate sleep disorders in children [53]. Thirty-three items within the following domains: bedtime resistance, sleep-onset delay, sleep duration, sleep anxiety, night wakening, parasomnias, sleep disordered breathing, and daytime sleepiness are rated on a 3-point scale. The CSHQ was proven to be reliable and valid [53].

#### 2.7.3. Quality of Life Assessment

Sleep-related quality of life evaluation will be performed using the 28-item parent-reported Child Health Questionnaire (CHQ). The CHQ is a valid and reliable tool to assess the impact of OSA on quality of life [54,55].

### 2.8. Intervention

#### 2.8.1. Oromyofunctional Therapy

All participants will receive an OMT program that consists of one individual session of 45 min per week for a period of 10 weeks and 10 min daily home practice. For children with Down syndrome, therapy duration is extended to 20 weeks, together with 10 min daily home practice [35]. The exercises are taught during the weekly sessions and practiced together with the child in a playful and guided manner. Subsequently, the child repeats the exercises daily at home according to the prescribed number of repetitions. Appendix A outlines which exercises are introduced in each week and subsequently practiced at home, both for non-syndromic children and children with Down syndrome, including the number of repetitions required for each exercise during home practice. Four therapeutic goals are selected based on literature and therapeutic experience to improve muscle resistance, balance the contraction of pharyngeal muscles, and correct abnormal functional and resting postures of the oropharyngeal structures in order to stabilize the upper airway during sleep [29,30,31,54,55,56]. The following therapy goals were established: Goal 1: Rehabilitation of nasal breathing; Goal 2: Stabilization of a closed mouth posture (competent lip seal) by correct position of jaw and lips; Goal 3: Stabilization of correct tongue posture, including differentiated tongue movement; Goal 4: Increasing strength and endurance of upper airway muscles. The specific exercises chosen to reach these goals were based on both evidence from the literature as well as insights from clinical experience [53,54,56,57,58]. Existing exercises were modified to ensure accessibility for young children and children with cognitive impairments. Adaptations included the incorporation of visual feedback components and enhancements to increase the exercises’ appeal and comprehensibility for the target population. An overview of all exercises selected and developed for each therapy goal is presented in Appendix A.

#### 2.8.2. Therapy Provider and Treatment Fidelity Checks

Therapy is provided by an SLP (J.V.) with experience in the diagnosis and treatment of OMDs in children. All therapy will be provided by the same SLP to avoid therapist effects. Treatment fidelity checks will be performed by two SLPs blinded to the research purpose. These SLPs will review video recordings of randomly selected therapy sessions taking into account an equal distribution between the groups.

### 2.9. Statistical Analysis

For the analysis of intervention effects, a paired sample t test (continuous, parametric), Wilcoxon signed rank test (continuous, nonparametric), or McNemar test (nominal) will be used. Intraclass correlation coefficient models and Cohen’s kappa will be used to determine inter- and intrarater reliability.

## 3. Discussion and Conclusions

OSA is a prevalent medical condition with significant implications for overall health and quality of life in the pediatric population. Early and effective treatment of OSA is crucial. However, current treatment methods are often invasive, insufficiently effective, or suffer from poor adherence. These challenges are even more pronounced in high-risk populations, such as children with Down syndrome, highlighting the need for alternative therapeutic strategies. Given the association between orofacial myofunctional disorders and OSA, OMT is emerging as a promising treatment for pediatric OSA.

Both objective measures (e.g., polysomnography, tongue strength) and subjective/patient-reported outcomes (e.g., sleep quality questionnaires) will be collected to assess the potential of OMT in treating pediatric OSA. Unlike previous pediatric studies, changes in OSA severity will not be limited to alterations in AHI alone. While the AHI is often used as the holy grail when it comes to grading OSA severity, it has become clear that the AHI has several inherent shortcomings and therefore it does not capture the full complexity of sleep-disordered breathing [32]. By evaluating a wide range of objective polysomnographic outcomes—including oxygen desaturation indexes, arousal indexes, sleep efficiency, and sleep architecture metrics—in addition to AHI, this study will provide a more comprehensive understanding of how OMT impacts OSA. In addition to objective measurements, validated questionnaires will be used to assess the impact of OSA on the child’s daily functioning and overall well-being. To the best of our knowledge, no previous studies have examined the effect of OMT as primary treatment for pediatric OSA. However, it can be assumed that replacing invasive surgical treatments, such as AT, with functional therapy would greatly benefit the child by eliminating the risk of surgical complications and exposure to anesthesia. Non-syndromic children without a history of AT will therefore also be included in the study. Another strength of this study is the implementation of a detailed therapy plan in which all therapeutic goals and exercises are described. This contrasts with previous research, where patients followed varying therapy programs with limited information on their content. Providing a comprehensive overview of the exercises used enhances reproducibility and facilitates both future research and clinical application. As for the design of the study, the efficacy of OMT will be evaluated in both non-syndromic children and children with Down syndrome by the use of a pretest–posttest design. While it is acknowledged that randomized controlled trials are often considered the gold standard from a methodological perspective, it is chosen not to adopt this design in the current study. There are both ethical and practical considerations that informed this decision. Ethically, exposing children to sham therapy or additional investigations without therapeutic benefit was deemed inappropriate, particularly given the vulnerability of the study population. Furthermore, given the complex and heterogeneous nature of OSA, especially in pediatric patients, a within-subject design allows us to better capture meaningful changes by comparing each child’s post-intervention outcomes to their own baseline performance. By not dividing the sample into treatment and control groups, we ensure that a larger proportion of children receive the actual therapeutic intervention. This is particularly valuable in a pediatric context, where reducing the burden of treatment and maximizing potential benefit is paramount. However, it must be acknowledged that the absence of a control group limits the inferential strength of the study. Without randomization, the design is susceptible to potential biases, including placebo effects and the influence of unmeasured confounders. Additionally, no formal sample size calculation could be performed for the Down syndrome group due to the exploratory and novel nature of this objective, which presents another methodological limitation. These factors should be considered when interpreting future findings, and they underscore the need for follow-up studies using more robust designs to confirm the efficacy of OMT in this population.

This protocol outlines a study designed to explore the potential of orofacial myofunctional therapy as a non-invasive, functionally targeted alternative or adjunct to conventional treatments for pediatric OSA, aiming to address the critical gap in current management strategies. It is hypothesized that OMT may improve sleep quality and overall well-being in children with OSA, including those with Down syndrome. This protocol provides a structured and transparent research framework that can serve as a foundation for future research in children with OSA or in other specific OSA populations with limited treatment options, such as children with Prader–Willy syndrome and adults with mild OSA or primary snoring. If the therapy is successful, the findings may contribute to a paradigm shift in the treatment of OSA by emphasizing the importance of functionally driven interventions.

## Figures and Tables

**Table 1:** Overview of components ENT examination.

**Inspection of the Face**
Facial morphological patterns [36] Gummy smile [37] Neck circumference at the level of cricothyroid cartilage [38,39]
Inspection of the nose
External examination: deformities, symmetry, size, and patency of nares, frontal and dorsal profile [40] Anterior rhinoscopy: septal deviations, mucosa, turbinate hypertrophy [40,41]
Inspection of the face
Tonsils: grading according to Friedman’s tonsil classification [42] Teeth: type of malocclusion (Angle’s classification: class I–III) Tongue: macroglossia, ankyloglossia, functionality [43,44] Mallampati score [45]

## Appendix A

**Table A1:** Instructions for performing the exercises included in the OMT program.

Exercise 1 Goal: 1,2 Timing NS: 1 w DS: 1–2 w	Smelling and humming	Become aware of the role of the nose by humming and smelling.	
Exercise 1 Goal: 1,2 Timing NS: 2–10 w DS: 4–20 w	Breathing through nose	Breathe in and out through your nose while your mouth is closed. (Gradually increase duration and awareness)	
Exercise 3 Goal: 1,2 Timing NS: 1 w DS: 1-2 w	Breathing through one nostril	Close your right nostril and breathe through your left for 1 min. Then, switch: Close your left nostril and breathe through your right for 1 min. (3×)	
Exercise 4 Goal: 1,2 Timing NS: 2–3 w DS: 4–6 w	Lippo	Put a plastic disk (Lippo) between your lips while performing another activity (e.g., drawing, coloring). (Gradually increase duration)	
Exercise 5 Goal: 1,2 Timing NS: 1–10 w DS: 1–20 w	Button	Put a button, to which a string is attached, behind the lips and pull the string in a forward direction with increasing force for 10 s (15×).	
Exercise 6 Goal: 1,2 Timing NS: 3–10 w DS: 6–20 w	Tape	Taping around the lips with stretching kinesiology tape (day and night training).	
Exercise 7 Goal: 3 Timing NS: 1–2 w DS: 1–4 w	Tongue tapping: alveolar ridge	With your mouth open, tap with the tip of your tongue against the alveolar ridge repeatedly, keeping your mandible fixed. (15×)	
Exercise 8 Goal: 3,4 Timing NS: 3–10 w DS: 6–20 w	Tongue pressure: alveolar ridge	With your mouth open, press the tip of your tongue against the alveolar ridge for 5 s, keeping your mandible fixed. (15×)	
Exercise 9 Goal: 3 Timing NS: 1–2 w DS: 1–4 w	Tongue tapping: up/down/left/right	Protrude your tongue to the maximum extension alternately: (1) upwards (2) downwards, (3) to the left, (4) to the right. (15×)	
Exercise 10 Goal: 3,4 Timing NS: 3–10 w DS: 6–20 w	Maximum tongue protrusion	Protrude your tongue to the maximum extension and hold it like that for 5 s: (1) upwards, (2) downwards, (3) to the left, (4) to the right (5×).	
Exercise 11 Goal: 3–4 Timing NS: 1–3 w DS: 1–6 w	Tongue clicking	With your mouth open, place your tongue tip on the alveolar ridge and suck your tongue against your palate. Quickly move your tongue downwards, making a clicking sound. (30×)	
Exercise 12 Goal: 3–4 Timing NS: 3–10 w DS: 5–20 w	Tongue suction	With your mouth open, place your tongue tip on the alveolar ridge and suck your tongue against your palate. Hold it like that for 5 s. (15×)	
Exercise 13 Goal: 1–2–3 Timing NS: 5–10 w DS: 6–20 w	Correct tongue posture	Close your mouth. Place your tongue tip on the alveolar ridge and suck your tongue against your palate. Breathe in and out through your nose. (Gradually increase duration and awareness)	
Exercise 13 Goal: 4 Timing NS: 3–10 w DS: 6–20 w	Tongue sliding	With your mouth open, place your tongue tip on the alveolar ridge and slide backward toward the soft palate. Keep your tongue in the posterior position for 5 s, then slide back forward. (5×)	
Exercise 14 Goal: 4 Timing NS: 3–10 w DS: 6–20 w	Expiratory effort: straw	Place the straw in your mouth, sealing your lips around it. Blow intensely through the straw for 5 s. (15×)	
Exercise 15 Goal: 4 Timing NS: 4–5 w DS: 7–8 w	Straw phonation	Place the straw in your mouth, sealing your lips around it. Blow through the straw while producing a sustained /u/ vowel for 5 s. (15×)	
Exercise 16 Goal: 4 Timing NS: 6–10 w DS: 9–20 w	Expiratory effort: straw in water	Place the straw in your mouth, sealing your lips around it. Submerge the end of the straw five centimeters into the water. Blow intensely through the straw for 5 s. (15×)	
Exercise 17 Goal: 4 Timing NS: 6–10 w DS: 9–20 w	Straw phonation in water	Place the straw in your mouth, sealing your lips around it. Submerge the end of the straw five centimeters into the water. Blow through the straw while producing a sustained /u/ vowel for 5 s. (15×)	
Exercise 18 Goal: 4 Timing NS: 1–2 w DS: 1–5 w	Expiratory effort: balloon	Breath in through your nose, breathe out while blowing the balloon. (15×) If the child is unable to blow a balloon, place the straw in your mouth, sealing your lips around it. Breathe out while blowing the balloon. (15×)	
Exercise 19 Goal: 4 Timing NS: 1–2 w DS: 1–5 w	Expiratory effort: blower ball	Place the blower in your mouth, sealing your lips around it. Blow intensely through the blower for 5 s, the ball goes up. (15×)	

Abbreviations: NS = non-syndromic; DS = Down Syndrome; w = week; × = repetitions; Goal 1: Rehabilitation of nasal breathing; Goal 2: Stabilization of a closed mouth posture (competent lip seal) by correct position of jaw and lips; Goal 3: Stabilization of correct tongue posture, including differentiated tongue movement; Goal 4: Increasing strength and endurance of upper airway muscles. Note: The exercises represent a combination of exercises drawn from existing literature on the effectiveness of OMT in OSA and [29,30,31,53,54,55,56,57,58].
